# ­2019 *PLOS Genetics* Research Prize: Fruit fly school – language and dialects for communicating a threat

**DOI:** 10.1371/journal.pgen.1008381

**Published:** 2019-09-12

**Authors:** Gregory S. Barsh, Gregory P. Copenhaver, Elapulli Sankaranarayanan Prakash, Daniela C. Zarnescu

**Affiliations:** 1 HudsonAlpha Institute for Biotechnology, Huntsville, Alabama, United States of America; 2 Department of Genetics, Stanford University School of Medicine, Stanford, California, United States of America; 3 Department of Biology and the Integrative Program for Biological and Genome Sciences, University of North Carolina at Chapel Hill, Chapel Hill, North Carolina, United States of America; 4 Department of Biomedical Sciences, Mercer University School of Medicine, Macon, Georgia, United States of America; 5 Departments of Molecular and Cellular Biology, Neuroscience and Neurology, University of Arizona, Tucson, Arizona, United States of America

Practicing and publishing science has many rewards, and one of the best is the opportunity to pause and recognize truly exceptional work done by members of your research community. It is in this spirit that we launched the annual *PLOS Genetics* Research Prize several years ago. The prize recognizes a paper published in the last year that is scientifically outstanding and has broad reach across the genetics community. Over 100 responses were received this year during a nomination process that is open to anyone. Selection of the prize recipient by the *PLOS Genetics* Editorial Board is based on several criteria including the number of independent nominations, number of citations, traditional and social media coverage, and most importantly assessment of the paper’s scientific strength and impact for the field. In addition to this year’s prize winner we would like to acknowledge two highly laudable nominations. The first is an article by Felix Key and colleagues which examined human genetic adaptation to ambient temperature and found strong evidence for positive selection in the TRPM8 cold receptor [1]. The editors thought this work was particularly important given the increasing challenges of global climate change. The paper is also notable in that it has been cited 9 times in the last year, has had over 13,000 page views and downloads, and was covered and shared extensively by social media and news outlets. The second is an article by María Angélica Bravo Núñez and colleagues that reveals novel evolutionary strategies employed by selfish genes that kill gametes that fail to inherit them [2]. In addition to being quickly cited by other papers and receiving media attention, many editors ranked this paper highly because understanding how meiotic drivers manipulate mechanisms of inheritance is a fundamental aspect of genetics.

The winner of the 2019 *PLOS Genetics* prize is an article by Balint Kacsoh, Julianna Bozler, and Giovanni Bosco [3]. This paper received attention in both the media and the scientific community, and, as described below by its editor and a nominator, was broadly interesting to our readership. We would also like to note, in the interest of transparency, that the senior author Dr. Bosco is a member of our Editorial Board, but that this did not influence the paper’s nomination, nor its evaluation by our Senior Editors during the final selection process.

Social learning occurs both within and between species and is essential for survival; communicating the location of food sources or threats has been recognized in a diversity of species including bacteria, plants, insects and mammals. Honeybees and ants are traditional models for social learning [4], but the powerful tools of fruit fly genetics have recently been brought to bear on these questions [5]. A central theme in these efforts is that genetic hardwiring and environmental cues interact to influence the acquisition and retention of new information in a social context, leading to physiological responses that affect behavior and survival [5,6]. In previous studies, the Bosco group showed that *Drosophila melanogaster* communicate threats to one another, within the same species, and trigger a physiological response resulting in fewer eggs being laid when parasitic wasps are present [5,7]. More specifically, female flies respond to the threat posed by wasps by depressing egg laying both acutely and long term, reflecting a memory of the threat. While previous studies established that exposed flies (“teachers”) can communicate threat to naïve flies (“students”) using only visual cues, it remained unknown if and how communication might extend to, or between, other fly species. In a remarkable *tour de force* involving 15 fly species separated by more than 30 million years of evolution, Kacsoh and colleagues [3] began answering these questions, and in the process, uncovered evidence for a fly “language” with multiple “dialects”.

The authors found that Drosophila species, closely and distantly related to *D*. *melanogaster*, exhibit a similar response to wasp threat that can be communicated between species. Perhaps not surprisingly, evolutionary distance is related to the extent of interspecies communication, efficient and extensive for closely related pairs (such as *D*. *simulans* and *D*. *melanogaster*), but impossible to detect for distantly related species (such as *D*. *melanogaster* and *D*. *virilis*). Furthermore, communicating wasp threat is hardwired, since the authors find that communication from a “teacher” to a conspecifc “student” raised in isolation is about as efficient as that between two closely related but different species. Surprisingly, when closely related Drosophila species inhabit the same container for a week, the extent of communication improves to the same level observed among conspecifics! So what happens during a week of cohabitation? The authors propose that this allows the animals to learn each other’s “dialects”, aided by socialization and bidirectional exchange of information. However, some very distantly related species such as *D*. *virilis* showed no ability to communicate with *D*. *melanogaster* even following cohabitation for a week. The authors also showed that when trained *D*. *melanogaster* is cohabitated with two other species capable of partial communication, flies are able to learn multiple dialects. Together, these results suggest that the ability to communicate threat is influenced both by genetics and environment and that the communication phenotype exhibits plasticity.

Does learning involve specific sensory modalities? Applying the awesome power of fly genetics, Kacsoh et al [3] discovered that when *D*. *ananassae* “students” were cohabitated with blind *D*. *melanogaster* mutant “teachers”, they could not learn the *D*. *melanogaster* dialect. This suggests that bidirectional, active learning is involved rather than mere mimicry. Furthermore, mutants with impaired wing movement or specific chemosensory signaling are unable to learn dialects. It will be interesting in the future to determine the exact molecule(s) required for dialect learning. In a recently published study, Kacsoh et al [8] further elucidate the neural circuitry mediating social learning in Drosophila. In keeping with previous findings that Orb2, a key memory gene is required in mushroom body (MB) neurons for intraspecies threat communication [5], the authors find that Orb2 and PTEN but not FMR1, another well-established learning and memory gene, are required for dialect learning [3]. These findings may have implications for a broader understanding of the relationship between learning and memory, extending to human conditions in which FMR1 affects behavior, communication, and learning [9].

The work by Kacsoh et al [3] cements Drosophila as an important genetic model system for investigating mechanisms that mediate dialect learning, social learning about specific environmental cues, and possible effects of learning on physiologic responses and behaviors. The approaches and findings will be of interest to ecologists, geneticists, neuroscientists, and psychologists that study all organisms.
